# Differential Toxicity of Water-Soluble Versus Water-Insoluble Components of Cowshed PM2.5 on Ovarian Granulosa Cells and the Regulatory Role of Txnip in Overall Toxicity

**DOI:** 10.3390/antiox15010138

**Published:** 2026-01-21

**Authors:** Zhenhua Ma, Xiqing Zhang, Xiaohui Du, Cuizhu Zhao, Yunna Jia, Ye Wang, Xintian Li, Xiuzhen Yu, Yunhang Gao

**Affiliations:** 1 College of Animal Science and Technology, Jilin Agricultural University, Changchun 130118, China; 2 Animal Husbandry and Veterinary Research Institute, Jilin Academy of Agricultural Sciences, Changchun 130033, China; 3 Shenyang Animal Disease Prevention and Control Center, Shenyang 110001, China; 4 Institute of Agricultural Mechanization, Xinjiang Academy of Agricultural Sciences, Wulumuqi 830091, China

**Keywords:** livestock farming environment, cowshed, PM2.5, water-soluble components, ovary, Txnip

## Abstract

Fine particulate matter (PM2.5)-induced ovarian damage has attracted widespread attention, but differences in cytotoxicity and underlying mechanisms of water-soluble (WS-PM2.5) and water-insoluble (WIS-PM2.5) fractions are unclear. To investigate potential effects of PM2.5 from livestock farming environments on animal ovaries, PM2.5 samples were collected from large-scale cattle barns. There were significant differences between fractions regarding elemental composition, proportion of water-soluble ions, polycyclic aromatic hydrocarbon content, and endotoxin concentrations. Based on transcriptome sequencing results, in a cowshed PM2.5 exposure model (rats), differentially expressed ovarian mRNAs were significantly enriched in signaling pathways such as cytokine interaction and the Hippo pathway, with the expression of thioredoxin-interacting protein (Txnip) significantly increased. In vitro (primary rat ovarian granulosa cells), short-term exposure to WS-PM2.5 (12 h) significantly induced inflammatory factor release, acute oxidative stress, mitochondrial dysfunction, and intracellular Ca^2+^ overload, with characteristics of rapid acute injury. However, extended (24 h) WIS-PM2.5 exposure had greater disruptive effects on estrogen homeostasis, intracellular enzyme release (LDH), and mitochondrial structure (subacute characteristics). Furthermore, downregulating Txnip expression via inhibitors effectively mitigated cowshed PM2.5-induced ovarian granulosa cell toxicity, oxidative stress, and mitochondrial and hormonal dysfunction. In summary, solubility of cowshed PM2.5 components affected cytotoxic characteristics, and Txnip was a key factor linking oxidative stress to granulosa cell damage. The study provided a mechanistic basis and potential targets for preventing and controlling PM2.5-induced ovarian damage in livestock environments.

## 1. Introduction

Fine particulate matter (PM2.5, aerodynamic diameter ≤ 2.5 μm) is an important air pollutant and a major factor contributing to various diseases [1], with PM2.5 adversely affecting the respiratory, cardiovascular, and reproductive systems [2,3]. As the respiratory system is the primary site of interaction with PM2.5, the impacts of PM2.5 on pulmonary diseases have been well-documented [4]. There are also associations between PM2.5 exposure and adverse reproductive outcomes in women, including increased miscarriage and preterm birth, as well as reduced fertility and diminished ovarian reserve [5,6]. Previous studies also demonstrated effects of respiratory exposure to PM2.5 on ovarian damage [7], implying PM2.5 can penetrate the respiratory system and affect other systems.

Ovarian health risks associated with PM2.5 are attributed to its complex and diverse chemical composition. The source, region, and season of PM2.5 sampling, and other factors (human activities, industrial types, air humidity, etc), significantly influence composition [8,9]; therefore, effects of PM2.5 on ovarian health may differ among environments. PM2.5 from livestock farming versus urban environments differs significantly in microbial and organic matter composition [10,11]. Several recent studies have quantitatively assessed PM2.5 emission levels across different types of livestock farms. Consistently, these findings have indicated that ambient PM2.5 concentrations in farm environments significantly exceed the established environmental safety thresholds, thereby highlighting the critical potential health risks posed by this pollutant to both livestock and farm workers [12,13]. In the livestock industry, PM2.5-induced damage to animal ovaries (e.g., cows and pigs) would have economic consequences [14]. Polycyclic aromatic hydrocarbons and heavy metals damage animal ovaries [15]. However, based on the complex and diverse chemical composition of PM2.5, focusing solely on the toxic effects of one component would be limiting. Additionally, the differing solubility of PM2.5 components affects their ability to cross the respiratory barrier and enter the bloodstream [16]. Therefore, comparing water-soluble (WS-PM2.5) and water-insoluble (WIS-PM2.5) fractions of PM2.5 should provide new information. However, the biological toxicity characteristics of WS-PM2.5 and WIS-PM2.5 in livestock environments are unknown.

Thioredoxin-interacting protein (Txnip) is a multifunctional protein with a key role in cellular stress response pathways. Its core structure has an α-arrestin domain, enabling interactions with thioredoxin (TRX), thereby inhibiting its antioxidant function and expression, ultimately increasing intracellular oxidative stress [17,18]. Under PM2.5 stimulation, Txnip is involved in forming the NOD-like receptor protein 3 (NLRP3) inflammasome complex, which is mediated by endoplasmic reticulum stress [19]. Furthermore, Txnip may regulate glucose and lipid metabolism via various pathways, e.g., modulation of hepatic gluconeogenesis, peripheral glucose uptake, lipogenesis, and substrate utilization [20]. In PM2.5-induced autophagy of vascular endothelial cells, Txnip participates in the initiation and formation of autophagosomes, with heavy metal components having a crucial catalytic role [21]. However, the status and function of Txnip within the ovary is are unclear. Therefore, this study focused on the relationship between Txnip and in vitro ovarian granulosa cell damage under the influence of PM2.5 in an animal husbandry environment.

Samples of PM2.5 were collected from representative large-scale cattle barns. The cytotoxic characteristics of WS-PM2.5 and WIS-PM2.5 were evaluated during in vitro exposure of ovarian granulosa cells. Additionally, the mechanism of Txnip in cowshed PM2.5-induced ovarian system damage was evaluated through ovarian transcriptomic analysis and in vitro experiments. These findings contribute to understanding the biological toxicity characteristics of cowshed PM2.5 and mitigating the risk of ovarian damage in animals.

## 2. Materials and Methods

### 2.1. Cowshed PM2.5 Sample Collection and Preparation

Detailed information on the cattle barn and PM2.5 collection process has been described [22]. In this study, the obtained PM2.5 samples were suspended in endotoxin-free ultrapure water and centrifuged at 3000 rpm for 1 h at 4 °C. The supernatant represented the water-soluble fraction, whereas the precipitate comprised the non-water-soluble particles; these fractions were freeze-dried to yield WS-PM2.5 and WIS-PM2.5, respectively [23,24]. For the study, both components were diluted in sterile PBS to the same concentration (5 × 10^3^ μg/mL). For in vitro experiments, DMEM medium was used to dilute both components to a final effective concentration of 50 μg/mL, which was demonstrated to be moderately effective in inducing cytotoxic responses in vitro. The exposure concentration of PM2.5 was determined based on the findings of previous studies and references to protocols employed in analogous research [7,25,26].

### 2.2. Physical and Chemical Properties of Cowshed PM2.5

The surface characteristics, microbial composition, and resistance gene composition of cowshed PM2.5 are well described [22,27]. In this study, further elemental analysis, aromatic hydrocarbon composition analysis, and water-soluble ion composition analysis were conducted on various components of cowshed PM2.5 samples. Water-soluble ions in WS-PM2.5 samples were assessed with ion chromatography at Shanghai Jiao Tong University. The types and concentrations of polycyclic aromatic hydrocarbons in WIS-PM2.5 samples were assessed using high-performance liquid chromatography. The elemental composition of WS-PM2.5 and WIS-PM2.5 samples was determined with inductively coupled plasma mass spectrometry.

### 2.3. Endotoxin Content Assay

The endotoxin content of WS-PM2.5 and WIS-PM2.5 samples was determined with an endotoxin detection kit (Toxin Sensor ^TM^ Chromogenic LAL Endotoxin Assay Kit, GenScript Biotech Corporation, Nanjing, China), in accordance with the manufacturer’s directions. Standards, test samples, and assay reagents were added to endotoxin-free tubes and incubated at 37 °C for 15 min, and the absorbance of each reaction was measured using a microplate reader at 545 nm. The endotoxin content of each sample was calculated based on a standard curve.

### 2.4. Transcriptomics and Data Analysis

Animal models exposed to cowshed PM2.5 have been detailed in previous studies [7]. Six-week-old (160–170 g) female Sprague-Dawley (SD) specific pathogen-free (SPF) rats (Liaoning Changsheng Biotechnology Co., Ltd., Benxi, China) were used for whole-body PM2.5 exposure modeling. Previous research has provided detailed descriptions of the exposure dose, exposure process, and parameters of the whole-body PM2.5 exposure apparatus [28]. The device effectively simulates the exposure of animals or humans to PM2.5 within the barn environment, thereby offering enhanced scientific reference data. Briefly, the rats were exposed to four times the actual ambient concentration of PM2.5 for 6 h a day for 30 days. The respiratory coefficient, single-breath volume, and respiratory frequency of adult rats were used to quantify their daily exposure to PM2.5. Rats living in clean air constituted the control group (Control) and received no additional treatment, whereas rats exposed to PM2.5 constituted the PM2.5 exposure group (PM2.5). In this study, ovarian samples were collected from rats in the Control and PM2.5 groups, respectively. After trimming off excess adipose and connective tissues, the fresh rat ovarian tissues were promptly placed into centrifuge tubes containing an RNA preservative, immediately snap-frozen in liquid nitrogen, and subsequently transferred to a −80 °C freezer for long-term storage. Three replicate samples from each group were selected and transferred to Hangzhou LC-Bio Technologies (Hangzhou) Co., Ltd. (Hangzhou, China) for analysis. The experiment was approved by the Jilin Agricultural University Animal Ethics Committee (Ethics No. 20231206001).

### 2.5. Culture and Grouping of Rat Ovarian Granulosa Cells

Methods for the extraction and identification of rat ovarian granulosa cells have been detailed in previous studies [7]. Rat ovarian granulosa cells were cultured in DMEM/F12 medium supplemented with 15% fetal bovine serum at 37 °C under 5% CO_2_. To investigate the cytotoxicity of WS-PM2.5 and WIS-PM2.5, rat ovarian granulosa cells were exposed to identical concentrations of each pollutant, with changes in cell viability monitored over a 24 h interval. Cells were divided into Control, WS-PM2.5, and WIS-PM2.5 groups. Additionally, to investigate the role of Txnip in PM2.5 toxicity, rat ovarian granulosa cells were divided into Control, Txnip inhibitor (T-IN), PM2.5, and PM2.5 + Txnip inhibitor (PM2.5 + T-IN) groups. In vitro experiments were conducted using a PM2.5 concentration of 50 μg/mL. The Txnip inhibitor, which has a recommended working concentration of 3 μM, was purchased from MCE (MedChemExpress LLC, Monmouth Junction, NJ, USA).

### 2.6. ELISA

Concentrations of cytokines, IL-1β (sensitivity: 1.56 pg/mL), IL-6 (sensitivity: 15.6 pg/mL), and TNF-α (sensitivity: 15.6 pg/mL), plus LDH (sensitivity: 2.51 ng/mL), were assessed with ELISA kits according to the manufacturer’s instructions (Jiangsu Meimian Industrial Co., Ltd., Yancheng, China).

### 2.7. Oxidative Stress Assessment

Commercial kits were used to measure superoxide dismutase (SOD) and glutathione peroxidase (GSH-PX) activity, as well as malondialdehyde (MDA) concentrations, according to the recommended protocol of the manufacturer (Nanjing Jiancheng Bioengineering Institute, Nanjing, China). Cells were harvested and lysed, and reagents were added. The OD values were then measured using a microplate reader and final concentrations of each indicator calculated. Concentrations of cellular reactive oxygen species (ROS) were measured using the 2′,7′-dichlorodihydrofluorescein diacetate (DCFH-DA) method. Under fluorescence microscopy, the intensity of green fluorescence corresponded to ROS concentrations.

### 2.8. ATP Assay

ATP concentrations were measured with a kit (#S0027, Beyotime Biotechnology, Shanghai, China) following the manufacturer’s guidelines. For this, RIPA buffer was used to lyse the cells, and then the lysate was centrifuged at 12,000 rpm for 5 min. The upper layer was collected and the BCA (Bicinchoninic Acid) method was used to determine protein content. For this, 20 μL of the sample was mixed with 100 μL of the assay working solution; relative light unit (RLU) was measured using a chemiluminescence detector (Atila Biosystems, Sunnyvale, CA, USA) and ATP concentration determined with a standard curve.

### 2.9. Intracellular Ca^2+^, JC-1 and MitoSOX Assays

Flou-4 AM, JC-1, and MitoSOX kits were purchased from Yeasen Biotechnology (Shanghai) Co., Ltd. (Shanghai, China). Each dye was incubated with cells subjected to various stimuli for 30 min. Unbound dye was washed away with PBS, and images were acquired under a fluorescence microscope (Olympus, Tokyo, Japan). Fluorescence intensity was measured using a fluorescence microplate reader (Spark, Tecan Group Ltd., Zurich, Switzerland).

### 2.10. qPCR and mtDNA Copy Number Assay

After collection and processing, cells were divided into two equal portions. A kit for DNA extraction (Sangon Biotech (Shanghai) Co., Ltd., Shanghai, China) was used to extract total cellular DNA from one portion to serve as the standardized control. The other portion was placed in a buffer containing 150 mM NaCl, 50 mM HEPES (pH 7.4), and 25 mg/mL digitalis. Cells were lysed at 4 °C for 10 min, then centrifuged at 980× *g* for 3 min, three times, to pellet cell debris. The resulting supernatant was centrifuged at 17,000× *g* for 10 min to obtain cytoplasm devoid of nuclei, mitochondria, and endoplasmic reticulum, and the DNA extraction kit was used to extract mitochondrial DNA (mtDNA). Subsequently, qPCR was performed using *COX1* as the mitochondrial-specific gene and the nuclear single-copy gene *GAPDH* from total cellular DNA as the internal control. Relative mtDNA content was calculated based on CT values (2^−ΔΔCT^). Primer sequences used in this study are listed in Table S1.

### 2.11. Western Blotting

Total proteins were extracted from tissues and cells using RIPA lysis buffer containing protease inhibitors. Proteins were separated by SDS-PAGE electrophoresis and subsequently transferred onto PVDF membranes (Millipore, Burlington, MA, USA). Membranes were blocked with 5% skim milk at room temperature for 2 h, followed by incubation with primary antibody at 4 °C. After 12 h, secondary antibody incubation was performed at room temperature for 2 h. Finally, after thorough washing with TBST solution, protein expression levels were assessed using the ECL chemiluminescent detection system (Monad Biotech Co., Ltd., Suzhou, China). Each Western blot experiment was performed in triplicate biological replicates.

The following primary antibodies were used: GAPDH and β-actin polyclonal antibodies (purchased from ABclonal Biotechnology Co., Ltd., Wuhan, China); and ATF6, CHOP, GRP78, Cyt-c, AhR, CYP1A1, and Txnip polyclonal antibodies (purchased from Proteintech Group, Inc., Wuhan, China). Secondary antibodies used were goat anti-rabbit or anti-mouse IgG, purchased from Proteintech Group, Inc., Wuhan, China. Detailed information regarding these antibodies is in Table S2.

### 2.12. Statistical Analysis

All measurements were repeated three times, and data were expressed as mean ± standard deviations (SD). Unpaired Student’s t-tests were used for pair-wise comparisons, whereas comparisons of three or more were performed with one-way analysis of variance (ANOVA) followed by Tukey’s multiple comparisons test using GraphPad Prism 8.0.1 software (GraphPad Software, Inc., San Diego, CA, USA). For all statistical analyses, *p* < 0.05 was considered statistically significant.

## 3. Results

### 3.1. Chemical Composition and Endotoxin Content Characteristics of Cowshed PM2.5

**Figure 1:**
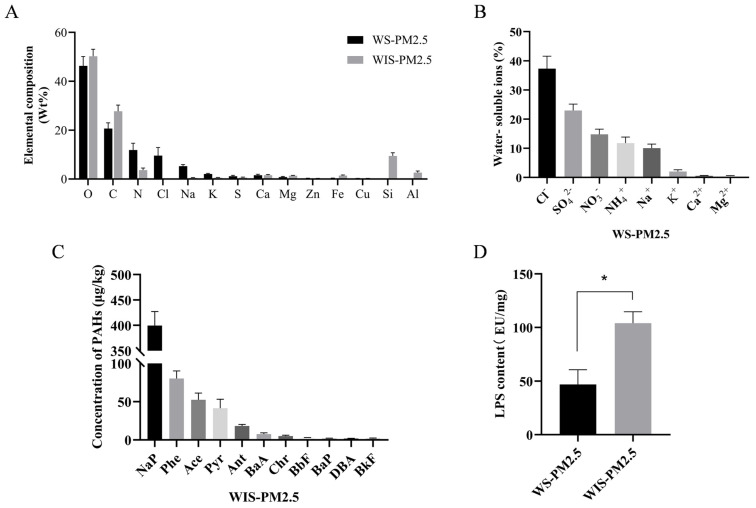
Chemical composition and endotoxin content characteristics of cowshed PM2.5. (**A**) Elemental composition analysis for cowshed PM2.5. (**B**) Analysis of water-soluble ion concentration proportion for cowshed PM2.5. (**C**) Analysis of polycyclic aromatic hydrocarbon concentration. (**D**) Endotoxin (LPS) content. * *p* < 0.05, compared to the WS-PM2.5 group.

Elemental analysis results for cowshed PM2.5 are in Figure 1A. In addition to the two fundamental elements O and C, WS-PM2.5 had relatively high proportions of N, Cl, Na, and K, whereas WIS-PM2.5 had relatively high proportions of Si, Al, Ca, and Mg. Predominant water-soluble ions in WS-PM2.5 were Cl^−^, SO_4_^2−^, NO_3_^−^, and NH_4_^+^ (Figure 1B).

Polycyclic aromatic hydrocarbons (PAHs) are poorly soluble substances. In WS-PM2.5, PAH concentrations did not reach the detection limit, whereas WIS-PM2.5 had abundant PAH content (Figure 1C). Among these, naphthalene (NaP), phenanthrene (Phe), anthracene (Ace), and pyrene (Pyr) were present at relatively high concentrations.

A key characteristic of PM2.5 samples collected from livestock areas, such as cattle barns, is their high microbial load. Endotoxin (LPS) was detected in both components, with higher LPS content in WIS-PM2.5 (Figure 1D). These findings suggest that the chemical composition of water-soluble and water-insoluble components in PM2.5 from cattle barns differs significantly in terms of LPS content, potentially exhibiting distinct biological toxicity profiles.

### 3.2. Cowshed PM2.5-Induced Differential Expression of Ovarian mRNAs in Rats

**Figure 2:**
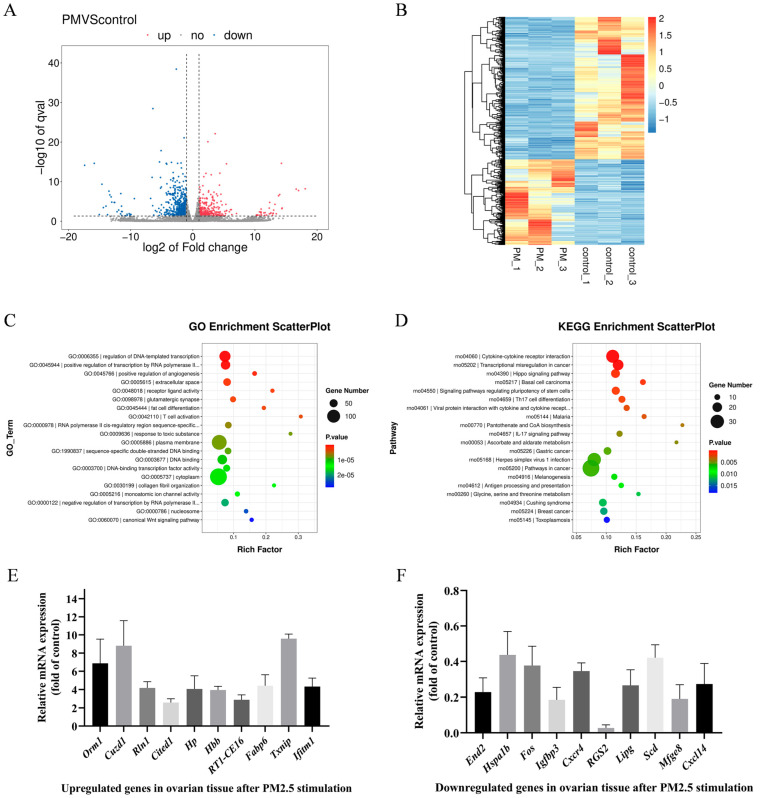
Cowshed PM2.5-induced differential expression of ovarian mRNAs in rats. (**A**) Volcano plot, number of differentially expressed genes. (**B**) Clustering analysis of differentially expressed genes. (**C**) GO enrichment analysis. (**D**) KEGG enrichment analysis. (**E**) qPCR validation results for upregulated genes. (**F**) qPCR validation results for downregulated genes.

To investigate potential biological toxicity mechanisms of PM2.5 in livestock farming environments on animal ovaries, RNA-Seq analysis was performed on rat ovarian tissue, with a total of 1095 differentially expressed genes (DEGs) in the PM2.5 group compared to the Control group. Among these genes, 410 were upregulated and 685 were downregulated (Figure 2A).

Hierarchical clustering analysis of DEGs revealed that expression trends in the PM2.5 group were nearly opposite to those in the Control group (Figure 2B). Additionally, in GO enrichment analysis, compared to the Control group, DEGs after PM2.5 exposure had the most significant enrichment in DNA template transcription regulation, RNA polymerase transcription regulation, angiogenesis regulation, receptor-ligand activity, and T-cell activation (Figure 2C). In KEGG enrichment analysis, compared to the Control group, PM2.5 exposure induced the most significant enrichment of DEGs in the following pathways: cytokine and cytokine receptor interactions, transcriptional dysregulation in cancer, Hippo signaling pathway, and stem cell signaling pathway (Figure 2D).

To validate the accuracy of the sequencing results, 10 significantly upregulated genes and 10 significantly downregulated genes were selected for qPCR validation, with gene expression trends consistent with sequencing data (Figure 2E,F). Overall, these findings demonstrate that PM2.5 exposure significantly alters ovarian gene expression and exerts a toxic effect that induces ovarian damage in rats.

### 3.3. Water-Soluble Components in Cowshed PM2.5 Had Short-Term Acute Cytotoxicity

**Figure 3:**
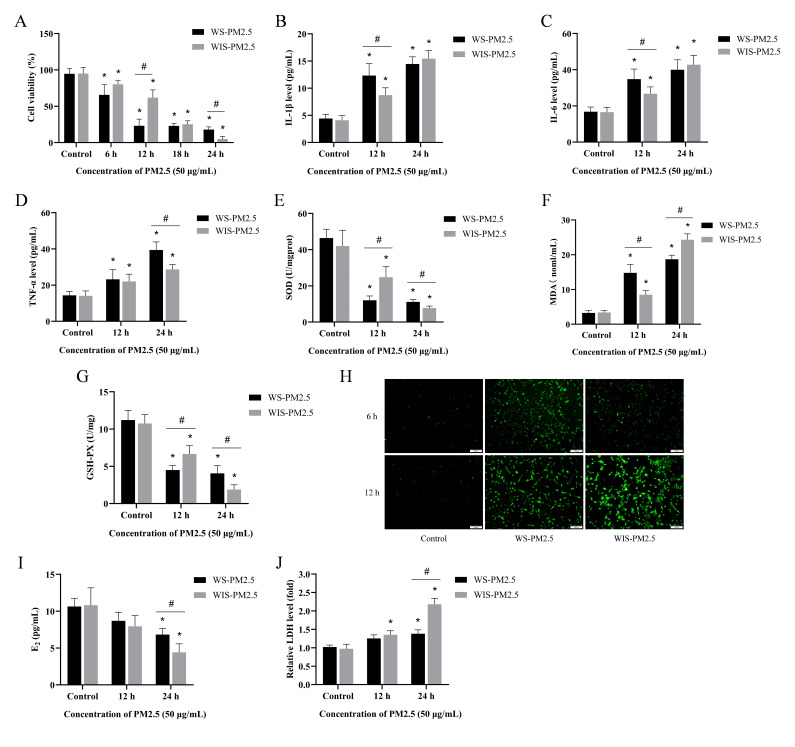
Water-soluble components in cowshed PM2.5 had short-term acute cytotoxicity. (**A**) Toxic effects of PM2.5 exposure on cells. (**B**–**D**) Concentrations of IL-1β, IL-6, and TNF-α in cells treated with PM2.5. (**E**–**G**) SOD, MDA, and GSH-PX in cells. (**H**) Cellular ROS fluorescence signal (green). Scale bar = 100 μm. (**I**,**J**) Changes in cellular E_2_ and LDH concentrations. * *p* < 0.05, compared to the Control group; ^#^ *p* < 0.05, compared to the WS-PM2.5 group.

Based on the cell viability assay, WS-PM2.5 induced a faster cytotoxic response compared to WIS-PM2.5 (Figure 3A). Regarding inflammatory cytokine responses, WS-PM2.5 also induced a more rapid expression of IL-1β, IL-6, and TNF-α in cells compared to WIS-PM2.5 (Figure 3B–D). Regarding oxidative stress, both water-soluble and water-insoluble components in PM2.5 induced oxidative stress in ovarian granulosa cells, including elevated ROS and MDA concentrations, as well as markedly decreased GSH-PX and SOD activity. Similarly, WS-PM2.5 exhibited more pronounced and rapid effects in the early stages of these processes. However, with prolonged exposure, WIS-PM2.5 exhibited a more pronounced trend (Figure 3E–H).

Both components induce alterations in E_2_ concentrations. For the first 12 h, there was no significant differences between the two components, whereas after 24 h, WIS-PM2.5 induced more pronounced hormonal changes (Figure 3I). WIS-PM2.5 induced a more pronounced release of LDH after a certain interval (Figure 3J). The above results indicate that both types of PM2.5 from cattle barns exert significant toxic effects on ovarian granulosa cells. WS-PM2.5 exhibits pronounced acute effects on cell viability, inflammatory response, and oxidative stress processes, while WIS-PM2.5 demonstrates greater significance in disrupting hormone expression and cell membrane permeability following prolonged exposure.

### 3.4. WS-PM2.5 Induced Rapid Responses in Mitochondrial Dysfunction and Endoplasmic Reticulum Ca^2+^ Release

**Figure 4:**
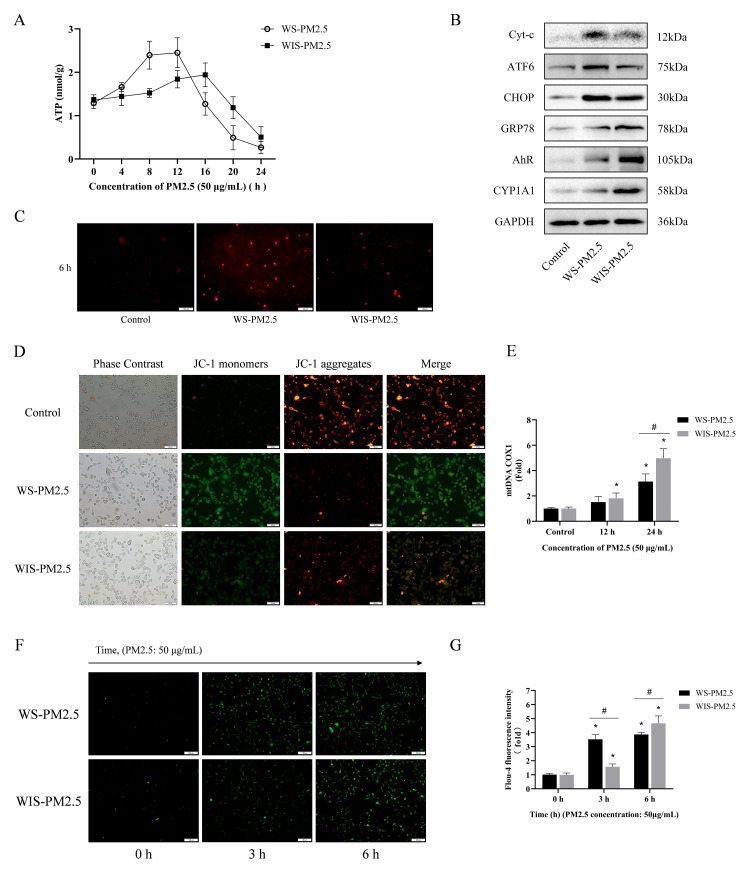
WS-PM2.5 induced rapid responses in mitochondrial dysfunction and endoplasmic reticulum Ca^2+^ release. (**A**) ATP concentrations in rat granulosa cells. (**B**) Detection of Cyt-c, ATF6, CHOP, GRP78, AhR, and CYP1A1 protein expression in rat granulosa cells. (**C**) Fluorescence staining of cellular mitochondrial superoxide MitoSOX. Scale bar = 100 μm. (**D**) Fluorescence staining of cellular mitochondrial membrane potential JC-1. Scale bar = 50 μm. (**E**) Cytoplasmic mtDNA content assay. (**F**,**G**) Fluo-4 AM staining of rat granulosa cells. Scale bar = 100 μm. * *p* < 0.05, compared to the Control group; ^#^ *p* < 0.05, compared to the WS-PM2.5 group.

Although previous studies have demonstrated that abnormalities in mitochondrial and endoplasmic reticulum function constitute key processes in cowshed PM2.5-induced cellular damage, the respective contributions of WS-PM2.5 and WIS-PM2.5 to this process remain unclear [28]. This study assessed the effects of both components on mitochondria and the endoplasmic reticulum. Regarding mitochondrial damage, WS-PM2.5 had a more rapid and pronounced impact on ATP concentrations (Figure 4A). After 12 h of WS-PM2.5 exposure, ATP concentrations peaked. Similarly, measurements of Cyt-c expression demonstrated acute effects of WS-PM2.5 (Figure 4B). The MitoSOX assay revealed that, within 12 h, WS-PM2.5 significantly increased cellular mitochondrial MitoSOX content (Figure 4C). JC-1 assays also showed the acute effects of WS-PM2.5 (see Figure 4D). Mitochondrial DNA (mtDNA) assays showed that both pollutants caused cytoplasmic migration of mtDNA, with WIS-PM2.5 resulting in greater leakage (Figure 4E). These results suggest that, compared to the acute effects of WS-PM2.5, WIS-PM2.5 causes more pronounced disruptions to cellular structure.

On the other hand, both components induced endoplasmic reticulum (ER) stress in ovarian granulosa cells, including increased expression of ATF6, CHOP, and GRP78 (Figure 4B). As a crucial intracellular calcium pool, the stability of [Ca^2+^]i (intracellular Ca^2+^) is essential for physiological processes of granulosa cells. Measurements of [Ca^2+^]i revealed that exposure to WS-PM2.5 for 3 h induced Ca^2+^ overload in granulosa cells (Figure 4F,G). Combined with the chemical composition analysis of PM2.5 (Figure 1), damaging effects of WS-PM2.5 on cell membranes may be associated with its relatively high content of PAHs. Therefore, changes in expression of aromatic hydrocarbon receptor-associated proteins (AhR and CYP1A1) were further examined at 24 h. WIS-PM2.5 exhibited more pronounced activation of the aromatic hydrocarbon receptor (Figure 4B). This may represent one of the key reasons for the differing cytotoxicity observed between the two components.

### 3.5. Inhibition of Txnip Alleviates Cowshed PM2.5-Induced Ovarian Granulosa Cell Toxicity

The results above indicate that oxidative stress (including mitochondrial oxidative stress) was a key manifestation of cowshed PM2.5-induced early acute damage to ovarian granulosa cells. Notably, transcriptomic sequencing revealed significantly elevated expression of *Txnip*. Therefore, this study used a Txnip inhibitor (Txnip-IN-1).

**Figure 5:**
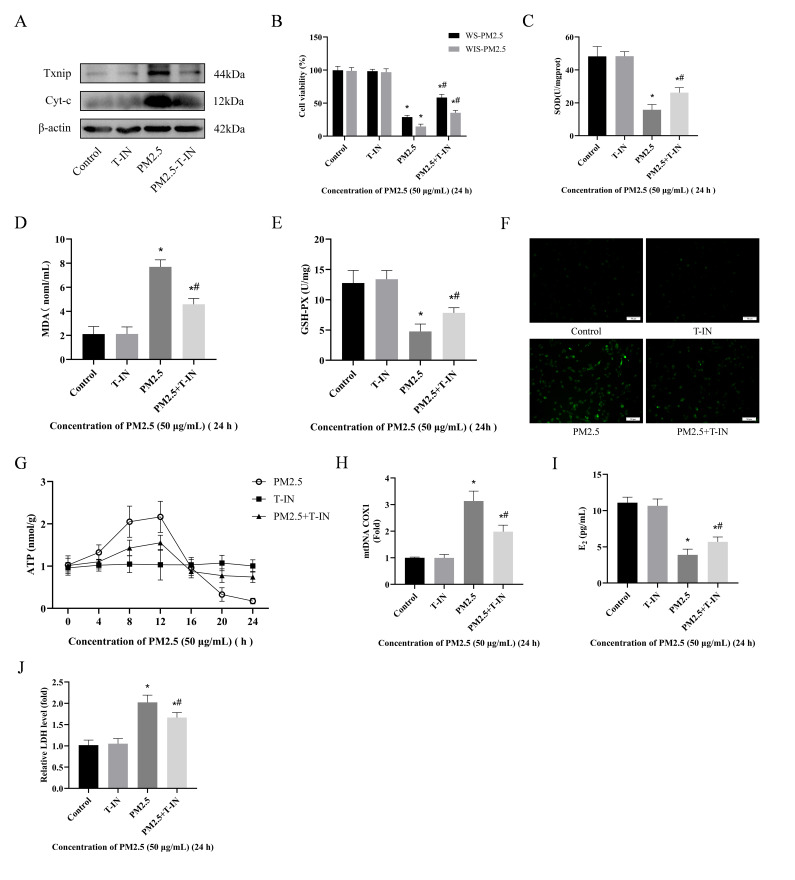
Inhibition of Txnip alleviated cowshed PM2.5-induced ovarian granulosa cell toxicity. (**A**) Detection of Txnip and Cyt-c protein expression in rat granulosa cells. (**B**) Changes in cell viability. (**C**–**E**) SOD, MDA, and GSH-PX concentrations in cells. (**F**) Cellular ROS fluorescence signal (green). Scale bar = 50 μm. (**G**) ATP concentrations in rat granulosa cells. (**H**) Cytoplasmic mtDNA content assay. (**I**,**J**) Changes in cellular E_2_ and LDH levels. * *p* < 0.05, compared with the Control group; ^#^ *p* < 0.05, compared to the PM2.5 group.

Subsequent to treatment with Txnip inhibitor, we re-evaluated the effect of cowshed PM2.5 on ovarian granulosa cells. Txnip-IN-1 effectively suppressed expression of Txnip protein (Figure 5A). Regarding cell viability, the PM2.5 + T-IN group significantly reversed ovarian granulosa cell viability compared to the PM2.5 group (Figure 5B). Regarding oxidative stress, the PM2.5 + T-IN group effectively reduced oxidative stress compared to the PM2.5 group, including decreased ROS and MDA concentrations and increased GSH-PX and SOD activities (Figure 5C–F). Regarding mitochondrial function, compared to the PM2.5 group, the PM2.5 + T-IN group mitigated changes in ATP fluctuations, mtDNA release, and Cyt-c expression (Figure 5A,G,H). Additionally, under the action of Txnip-IN-1, expression levels of E2 and LDH tended toward stability (Figure 5I,J). These results indicated that Txnip inhibitors can effectively suppress cowshed PM2.5-induced damage to ovarian granulosa cells.

## 4. Discussion

Elemental analysis of cowshed PM2.5 revealed exceptionally abundant nitrogen sources in WS-PM2.5, including urea ([NH_2_]_2_CO) from urine, ammonium salts (NH_4_^+^) produced by fecal decomposition, and soluble nitrates (NO_3_^−^) from feed [29,30]. These nitrogen-containing compounds have extremely high-water solubility, consistent with nitrogen’s relatively high elemental content among water-soluble components. The primary reason for the relatively high proportion of the elements Cl, Na, and K is probably their presence in water-soluble chloride forms (NaCl and KCl). Sources include salt additives in feed, as well as metabolic by-products in animal sweat and urine [31]. WIS-PM2.5 had elevated Si and Al concentrations, likely originating from aluminosilicate minerals in soil particles and bedding materials, serving as core elements of non-water-soluble mineral components. Relatively high proportions of Ca and Mg were mainly attributed to their presence as insoluble carbonates and phosphates within non-water-soluble particles. Additionally, calcium and magnesium supplements in feed are another likely source [32,33]. Transition metals have a strong inhibitory effect on cell viability [34]. Among water-soluble components, NH_4_^+^ originates primarily from the decomposition of urea in urine and microbial degradation of nitrogen-containing organic matter in manure. Within cattle housing environments characterized by extremely high production rates and exceptional water solubility, it is the predominant water-soluble nitrogen ion, thus constituting the largest proportion among water-soluble ions. There was a strong link between SO_4_^2−^ and NH_4_^+^ and production of ROS [35].

Analysis of PAHs in WIS-PM2.5 identified naphthalene (NaP) as the most prevalent compound. Naphthalene has many sources, with combustion of fossil fuels and biomass being major contributors. Furthermore, certain pesticides, preservatives, wood preservatives, and plastic treatment agents may contain naphthalene, which can slowly be released into the environment [36]. Naphthalene may affect the reproductive system, leading to reduced sperm count and fetal developmental abnormalities (e.g., deformities) [37].

WS-PM2.5 and WIS-PM2.5 are two distinct chemical substances, both with the potential to interact with host cells. However, WS-PM2.5 more readily enters cells and rapidly induces early adverse reactions, triggering oxidative stress and inflammatory damage [38]. In contrast, WIS-PM2.5 causes physical damage to cell membranes when absorbed by cells through active processes and serves as a carrier for WS components, having a crucial role in inflammatory responses [23,39]. These processes reduce cellular vitality and may impair associated functions. Therefore, it is crucial to understand the variability of PM2.5 cytotoxicity across locations and seasons to elucidate toxicological effects and mechanisms.

Transcriptome sequencing provides a comprehensive reflection of the relationship between mammals and environmental toxins [40], investigating pathogenic mechanisms and identifying therapeutic targets. Exposure to cowshed PM2.5 induced significant downregulation of the *RGS2* (Regulator of G protein Signaling 2) gene. In previous studies conducted by our research group, *RGS2* was also significantly downregulated in lung tissue following PM2.5 exposure [7,22]. We inferred that systemic regulatory pathways may be involved in the process by which PM2.5 damages to organs. Additionally, among all significantly upregulated DEGs, *Txnip* exhibited the most prominent expression alteration, implying that it may play a crucial role in rat ovarian injury induced by cowshed PM2.5. Thus, the present study focused on investigating the function of *Txnip*. Besides Txnip, *Cuzd1* (CUB and zona pellucida-like domains 1) also showed a high expression level (Figure 2E). A clinical serum screening study revealed that serum *Cuzd1* levels are significantly elevated in ovarian cancer patients, supporting *Cuzd1* as a novel serum biomarker for this disease [41]. The marked upregulation of *Cuzd1* strongly suggests that PM2.5 in livestock environments exerts adverse effects on animal ovaries.

In the GO functional enrichment analysis, receptor–ligand interactions and T-cell activation were significantly enriched following PM2.5 exposure. The physiological activities of the ovaries involve complex hormonal responses and regulation. Significant alterations in receptor–ligand activity may represent a key factor in ovarian hormonal disorders and premature ovarian failure [42]. Furthermore, T-cell activation has clinical importance in the evaluation of ovarian tumors [43]. Changes in T-cell subsets and T-cell content may indicate impaired immune killing function and shifts in immune tolerance within ovaries, which are closely associated with the development and progression of ovarian tumors, as well as immune escape mechanisms [44].

KEGG enrichment analysis revealed significant enrichment in signaling pathways, including those involving cytokine and cytokine receptor interactions, as well as the Hippo pathway. Within the cytokine and cytokine receptor interaction process, extensive changes in cytokine concentrations were implicated, including chemokines and inflammatory cytokines. We inferred that cowshed PM2.5 triggers frequent cytokine activity in the ovaries. In mammals, additional membrane protein receptors located upstream of the Hippo signaling pathway detect growth-inhibitory signals from the extracellular environment. Through a series of phosphorylation reactions catalyzed by kinases, these receptors ultimately interact with cytoskeletal proteins, regulating organ size and volume [45,46]. Following PM2.5-induced ovarian damage, changes in the size of ovarian organs may be associated with alterations in the Hippo signaling pathway. This pathway comprises multiple components that influence cellular fate and function in the ovary by regulating processes such as steroid hormone synthesis, primordial follicle activation, and follicular growth and development [47]. Given the critical role of these processes in the ovary, dysregulation of the Hippo signaling pathway inevitably leads to loss of ovarian follicular homeostasis, resulting in reproductive disorders such as polycystic ovary syndrome (PCOS), premature ovarian insufficiency (POI), and ovarian tumors [46]. The Hippo signaling pathway was significantly enriched, providing compelling evidence for the detrimental effects of cowshed PM2.5 on ovarian function.

Both cowshed PM2.5 components had distinct “acute-subacute” toxicity profiles toward ovarian granulosa cells. WS-PM2.5 primarily exhibits an “acute rapid response”: it can cause damage within a short interval via decreased cell viability, inflammation initiation, early oxidative stress, acute mitochondrial dysfunction, and calcium overload. This may be related to its water-soluble nature, as water-soluble components more readily and rapidly penetrate cell membranes to enter cells, directly acting on intracellular targets (e.g., mitochondria and ER calcium channels) to trigger immediate stress responses. Notably, the higher LPS content detected in WIS-PM2.5 is inconsistent with the stronger acute toxicity exhibited by WS-PM2.5. This discrepancy may be attributed to the different existing forms of LPS in the two fractions: LPS in WIS-PM2.5 is mainly associated with bacterial cells, whereas that in WS-PM2.5, albeit lower in concentration, predominantly exists in the form of free small molecules. Compared with cell-bound LPS, free LPS possesses higher efficiency in binding to cell surface receptors and penetrating cell membranes to enter the cytoplasm [48]. The present study did not conduct an in-depth investigation into the effect of existing LPS forms on its biological activity, which is a limitation of this work. Future studies will carry out dedicated experiments to address this key scientific issue.

In contrast, WIS-PM2.5 primarily caused “subacute cumulative damage”. Long-term exposure has a more pronounced effect on areas such as hormonal secretion disruption (E_2_), damage to cell membrane integrity (LDH release), and mitochondrial structural damage (mtDNA leakage). Non-water-soluble components, such as lipophilic substances like polycyclic aromatic hydrocarbons, may gradually accumulate within cellular or subcellular structures, such as cell and organelle membranes. This disrupts cellular structural and functional homeostasis, causing long-term toxicity [49,50]. Ovarian granulosa cells are the primary site for E_2_ synthesis, and this process relies on a critical catalytic reaction mediated by aromatase (CYP19A1). A key underlying mechanism may be that organic compounds such as PAHs in WIS-PM2.5 can activate the aryl hydrocarbon receptor (AhR) pathway, compete with estradiol for binding to estrogen receptors, and downregulate *CYP19A1* gene expression, thereby inhibiting E_2_ synthesis at the transcriptional level [51,52]. We inferred that the toxic mechanisms of both components were closely related to disruptions in mitochondrial, endoplasmic reticulum, and Ca^2+^ homeostasis. A dual pattern of mitochondrial damage emerged: WS-PM2.5 primarily interfered with mitochondrial function (energy metabolism and oxidative stress), whereas WIS-PM2.5 tended to damage mitochondrial structure (mtDNA leakage). Mitochondria serve as the core of cellular energy metabolism, and damage to their function or structure directly leads to reduced cellular vitality. Concurrently, mtDNA leakage may further activate cytoplasmic inflammatory pathways (such as cGAS-STING), exacerbating chronic inflammatory responses [53,54]. Conversely, ER stress and calcium overload have an early driving role; WS-PM2.5 rapidly induced both of them.

Calcium homeostasis imbalance is a key regulator of granulosa cell functions, such as hormone synthesis; early calcium overload may suppress cellular vitality and trigger inflammation by acting as an upstream event [55]. Additionally, differential activation of the aromatic hydrocarbon receptor pathway occurs. Given that WIS-PM2.5 may be rich in polycyclic aromatic hydrocarbons, it exhibits more pronounced activation of AhR/CYP1A1. This pathway may amplify acute toxic effects by regulating expression of inflammatory mediators and genes associated with oxidative stress.

Oxidative stress, particularly mitochondrial oxidative stress, was a key feature of the early acute damage caused to ovarian granulosa cells by PM2.5. Significantly elevated expression of Txnip, as revealed by transcriptomic sequencing, implied that it may have a pivotal role in linking oxidative stress to cytotoxicity. Txnip was significantly annotated in upregulated genes, consistent with the oxidative stress response. Consequently, the present study further validated the pivotal role of Txnip in the PM2.5 toxicity pathway by conducting intervention experiments using the Txnip-specific inhibitor (Txnip-IN-1). Txnip-IN-1 is specifically engineered to target the binding interface between Txnip and thioredoxin (Trx). This induces a steric hindrance effect to block the Txnip-Trx interaction, without impairing the intrinsic catalytic activity of Trx, which constitutes the core molecular basis for its targeting specificity. In contrast to other inhibitors, Txnip-IN-1 exerts a direct effect on the Txnip protein itself, thereby avoiding off-target interference with other signaling pathways. Consistent with this, Txnip-IN-1 has been directly utilized as a targeted inhibitor of Txnip in multiple previous studies [56,57]. This provided crucial evidence for elucidating the toxicity mechanism and identifying potential intervention targets. From the perspective of changes in cellular vitality, the decreased viability of ovarian granulosa cells induced by cowshed PM2.5 exposure was significantly reversed following Txnip-IN-1 intervention (Figure 5B). This suggests that abnormal Txnip activation had a pivotal role in the reduction in cellular survival capacity induced by PM2.5, phenotypically consistent with the acute oxidative stress-induced damage to cell viability observed in the WS-PM2.5 group.

Additionally, Txinp-IN-1 significantly alleviated the oxidative stress induced by cowshed PM2.5. It also mitigated abnormal E_2_ hormone secretion, fluctuations in ATP concentrations, and increased LDH release. Txnip activation is typically closely associated with intracellular stress signaling pathways [58]. In this study, PM2.5 induced endoplasmic reticulum stress (evidenced by increased expression of ATF6, CHOP, etc.). Txnip participated in the toxicity cascade through endoplasmic reticulum–mitochondrial crosstalk regulation. Meanwhile, some studies suggest that the interaction between Txnip and thioredoxin influences the activity of intracellular, redox-sensitive signaling molecules, e.g., NF-κB. This regulates the expression of inflammatory cytokines, such as IL-1β and IL-6 [59,60]. This provides a potential mechanistic explanation for the dual effects of Txnip-IN-1 on improving cellular viability and inflammatory responses.

This study had certain limitations. Firstly, the PM2.5 samples used were collected from large-scale cattle barns. Consequently, results were not fully representative of, or applicable to, the toxicity of PM2.5 in other livestock environments, such as pig or chicken barns. Therefore, future studies should conduct comprehensive comparative research on various types of livestock environments and factors such as seasonal variations. In addition, this study focused solely on the single regulatory role of Txnip. Future research will progressively explore its interactions with other pathways, such as the Hippo signaling pathway identified through transcriptomic analysis and the association mechanisms between cytokine interaction pathways and Txnip. Through subsequent studies, we will progressively construct and enrich the comprehensive molecular network encompassing “component differences-signaling pathway crosstalk-toxicity differentiation.”

## 5. Conclusions

This study focused on the toxic mechanisms of cowshed PM2.5 on ovarian granulosa cells. Due to differences in component solubility, WS-PM2.5 and WIS-PM2.5 exhibited “acute-subacute” differentiated biological toxicity characteristics with respect to inflammatory factor release, oxidative stress, mitochondrial function, [Ca^2+^]i, E_2_ disruption, and intracellular enzyme leakage. Transcriptome sequencing revealed differential expression of Txnip in ovaries. In vitro experiments demonstrated that Txnip was involved in processes such as oxidative stress, and its inhibitor effectively mitigated PM2.5-induced cytotoxicity in cattle barns. This study provided a mechanistic basis and potential targets for preventing and controlling PM2.5-induced ovarian damage in livestock environments.

## Data Availability

The original contributions presented in this study are included in the article and Supplementary Materials. Further inquiries can be directed to the corresponding authors.

## Supplementary Materials

The following supporting information can be downloaded at https://www.mdpi.com/article/10.3390/antiox15010138/s1.
