# Supplementary material for: Differential Toxicity of Water-Soluble Versus Water-Insoluble Components of Cowshed PM2.5 on Ovarian Granulosa Cells and the Regulatory Role of Txnip in Overall Toxicity
Source: Antioxidants (Basel). 2026 Jan 21;15(1):138. doi: 10.3390/antiox15010138 (PMC12837696; doi:10.3390/antiox15010138)
Supplement: Supplementary file 1 [file antioxidants-15-00138-s001.zip › Supplementary-Table S1.pdf]

Table S1. qPCR Primer Sequences

Gene	Forward Primer (5′→3′)	Reverse Primer (5′→3′)
Orm1	GCCAACATCACCCTAGGCAT	GAACTGCCTGCTTGAACACG
Cuzd1	GCGGGTGTAGTCGAGATGAG	GCCTTGATTGCAGCGAGATG
Rln1	CAGCTGGGTGAAGCAGAAGA	TCTGGTACAACCGATGTGGC
Cited1	CTGCACTGGATGTCAAGGGT	TTGGCTTTGGCTCCATTTGC
Hp	AGTGAGAATGCGACAGCCAA	CCCGATGTCCACCACAGAGC
Hbb	TGCACCTGACTGATGCTGAG	CCTCTGGGTCCAAGGGTAGA
RT1-CE16	AGATCCCCCAAAGGCACATG	ATTCAACTGCCAGGTCAGGG
Fabp6	AGGCAACCGTGAAGATGGAG	TAGGTCACATCCCCGATGGT
Txnip	AGTTACCCGAGTCAAAGCCG	CCAAGGTCTGTTTGCACTGC
Ifitm1	CTGAGATCTCCACGCCTGAC	CACCCACCATCTTCCTGTCC
End2	ACTAGCAAGACGTGGACTGC	CTCTTGTCAACTCTGGCCGT
Hspa1b	AGTCGGACATGAAGCACTGG	AGTCGGACATGAAGCACTGG
Fos	GGGAGCTGACAGATACGCTC	ATTGGCAATCTCGGTCTGCA
Igfbp3	AACAGTGTCGCCCTTCCAAA	CTTGGTGTCATAGCCTGGCA
Cxcr4	GGCTACCGTATTACGTGGGG	AACAGTGGAAGAAGGCGAGG
RGS2	TTCATCGAGAAGGAAGCTCCCAAAG	AAGCAGCCACTTGTAGCCTCTTG
Lipg	GGGTGGACATCAACAGGAGG	GACATCGTTGAATCCGCAGC
Scd	GGTATCGCCCCTACGACAAG	TAGGGGAAGGCGTGATGGTA
Mfge8	ATCTACTGCCTCTGCCCTGA	AAGCAAGGGTTTGGGGAACA
Cxcl14	AAAGTACCCACACTGCGAGG	TCTCGTTCCAGGCGTTGTAC
GAPDH	CCTGCACCACCAACTGCTTA	CATCACGCCACAGCTTTCCA
