# Supplementary material for: Differential Toxicity of Water-Soluble Versus Water-Insoluble Components of Cowshed PM2.5 on Ovarian Granulosa Cells and the Regulatory Role of Txnip in Overall Toxicity
Source: Antioxidants (Basel). 2026 Jan 21;15(1):138. doi: 10.3390/antiox15010138 (PMC12837696; doi:10.3390/antiox15010138)
Supplement: Supplementary file 1 [file antioxidants-15-00138-s001.zip › Supplementary-Table S2.pdf]

Table S2. Details of antibodies used in the Western blot.

Antibody	Item No.	Dilution	Manufacturer
GAPDH	A19056	1:50000	ABclonal
β-actin	AC026	1:50000	ABclonal
ATF6	24169-1-AP	1:1000	Proteintech
CHOP	15204-1-AP	1:1000	Proteintech
GRP78	11587-1-AP	1:1000	Proteintech
Cyt-c	10993-1-AP	1:5000	Proteintech
AhR	28727-1-AP	1:1000	Proteintech
CYP1A1	13241-1-AP	1:1000	Proteintech
Txnip	18243-1-AP	1:1000	Proteintech
Goat Anti-Rabbit IgG	SA00001-2	1:10000	Proteintech
Goat Anti-Mouse IgG	SA00001-1	1:10000	Proteintech
